# O07 The economic and clinical burden of antibiotic-resistant community-acquired *Escherichia coli* urinary tract infections

**DOI:** 10.1093/jacamr/dlaf230.007

**Published:** 2025-12-04

**Authors:** Emma Rose Carter, Paul Aylin, Kavitha Saravanakumar, Raheelah Ahmed, Alison Holmes, Nina Zhu

**Affiliations:** Health Protection Research Unit, Imperial College London, London, UK; Health Protection Research Unit, Imperial College London, London, UK; NHS North West London CCG, London, UK; Health Protection Research Unit, Imperial College London, London, UK; City University, London, UK; Fleming Initiative, Imperial College London, UK; Health Protection Research Unit, Imperial College London, London, UK; Fleming Initiative, Imperial College London, UK; Health Protection Research Unit, Imperial College London, London, UK; City University, London, UK; Fleming Initiative, Imperial College London, UK

## Abstract

**Background:**

Antibiotic resistance increases healthcare usage and the economic burden of infection.

**Objectives:**

To quantify the clinical burden and economic costs associated with antibiotic resistance in community-acquired *Escherichia coli* urinary tract infections.

**Methods:**

We identified *E. coli* urinary tract infections using linked microbiology data and GP clinical records from the Whole System Integrated Care (WSIC) dataset between September 2018 and July 2023 in Northwest London (NWL). These were defined as positive *E. coli* urine cultures with an associated UTI prescription, a lower UTI diagnosis, or symptoms (±3 days for community cultures; -3 to 0 days for hospital cultures). UTI episodes were excluded for cases with incomplete follow-up, hospital-associated infections (admission within 30 days prior) and incomplete information (no resistance testing). Clinical outcomes were tracked over the following 60 days and costed using relevant literature, including community-based GP encounters (PSRU - Unit Costs of Health and Social Care), antibiotic prescriptions (NHS drug tariff), urinary diagnostics (literature) and secondary care activities: UTI-related hospital admissions, A&E attendances and outpatient activity. All secondary care costs were calculated using the NHS tariff from Health-Related Group Costings. Costs were uprated to 2023. Univariate analysis of health outcomes was conducted using the chi-squared test. A generalized linear model was employed to assess the effect of resistance, adjusting for confounders, on total medical costs and GP encounters, using gamma and negative binomial distributions, respectively, in a propensity score-matched (PSM) cohort. Costs were winsorized at the 95% quantile due to right skew. Cohorts were matched based on previous antibiotic use, prior hospital admissions, ethnicity, IMD quintile, sex and age. Resistance of interest included trimethoprim, nitrofurantoin, ciprofloxacin, third-generation cephalosporin (3GC) and MDR. Average marginal effects were derived from the GLM models and applied to the resistant cohort to estimate the total cost associated with resistance.

**Results:**

In our cohort, we identified 29 686 *E. coli* UTI infections in 25 032 patients. Overall, additional GP visits and UTI-related hospital admissions were observed in 59.4% (17 645/29 686) and 3.5% (1038/29 686) of UTI episodes, respectively. The median [IQR] total medical cost of *E. coli* infection was £112 [£63–£174]. All infections cost a total of £9 059 158 in NWL over the study period. From the PSM matched cohort (Trimethoprim: S: 8465, R: 8465; Ciprofloxacin: S: 3171, R: 3171; MDR: S: 1178, R: 1178; 3GC: S: 2471, R: 2471), trimethoprim, third generation cephalosporins and ciprofloxacin were associated with higher proportions of UTI-related hospital admissions (*P<*0.05). All antibiotic resistance was linked to increased rates of GP utilization (*P<*0.05). Antibiotic resistance was associated with 42%, 36%, 29% and 28% higher total medical costs in episodes with ciprofloxacin, MDR, third-generation cephalosporin and trimethoprim resistance, respectively, compared to susceptible infections (*P<*0.05). The excess total medical costs associated with trimethoprim resistance were £41 per *E. coli* infection, resulting in a total excess cost of £349 858 in NWL (*n*=8508).

**Conclusions:**

This cost-of-illness study provides further evidence that the economic burden of resistance cannot be understated. This analysis included *Klebsiella pneumoniae* and *Pseudomonas aeruginosa*.

